# Kidney transplant outcomes in HLA desensitized patients with pretransplant CDC and/or FCM positive crossmatches

**DOI:** 10.3389/fimmu.2025.1612462

**Published:** 2025-06-23

**Authors:** Johan Noble, Céline Dard, Diane Giovannini, Hamza Naciri Bennani, Pierre Fournier, Béatrice Bardy, Anne Bourdin, Farida Imerzoukene, Lionel Motte, Florian Terrec, Paolo Malvezzi, Thomas Jouve, Lionel Rostaing

**Affiliations:** ^1^ Nephrology, Hemodialysis, Apheresis and Kidney Transplantation Department, Grenoble University Hospital, Grenoble, France; ^2^ Univ. Grenoble Alpes, Grenoble, France; ^3^ HLA Laboratory, Etablissement Francais Du Sang (EFS), La Tronche, France; ^4^ Histopathology Laboratory, Grenoble University Hospital, Grenoble, France

**Keywords:** HLA incompatible transplantation, kidney transplantation, immunoadsorption, positive CDC crossmatch, desensitization, cPRA, highly sensitized patients

## Abstract

**Background:**

Kidney transplant (KT) candidates with very high calculated panel reactive alloantibody (cPRA >95%) have limited chances to receive an HLA-matched transplant unless they undergo pretransplant desensitization.

**Objective:**

To assess the efficacy of immunoadsorption (IA) in desensitizing pretransplant KT candidates with high cPRA and positive crossmatch.

**Materials and methods:**

This was a single-center retrospective cohort study involving highly HLA-sensitized patients (cPRA >85%). Forty-nine patients underwent HLA-incompatible (HLAi) KT, of whom 25 (51%) received kidneys from deceased donors. Of these 49 patients, 23 had either a positive complement-dependent cytotoxic cross-match (CDC) and/or a positive flow cytometry cross-match (FCM). The remaining 26 patients had donor-specific anti-HLA (DSAs) detectable only by Luminex (CDC and FCM cross-matches were negative). Only CDC-positive and FCM-positive patients were desensitized. These 49 patients were compared with 160 patients who had cPRA >85% but underwent HLA-compatible (HLAc) KT, i.e., without pretransplant DSAs.

Pretransplant desensitization included IA sessions, rituximab, tacrolimus, steroids, and mycophenolate mofetil. Induction therapy consisted of antithymocyte globulins.

**Results:**

The mean follow-up duration was 7.4 ± 4 years. At 1-year and at last follow-up, 43 patient and death-censored graft survival rates were similar between HLAc and HLAi patients. However, HLAi patients experienced significantly more biopsy-proven rejections compared to HLAc patients. These rejections were predominantly antibody-mediated. Finally, the rate of infectious complications was similar between HLAc and HLAi patients.

**Conclusion:**

IA in addition to immunosuppression is an effective option for desensitizing HLAi patients, yielding favorable long-term outcomes.

## 1 Introduction

Kidney transplantation (KT) remains the preferred therapeutic approach for patients with end-stage kidney disease. Nevertheless, humoral alloimmune responses against human leukocyte antigens (HLA) represent the major obstacle to successful KT. Presently, approximately 30–40% of patients on KT waiting lists worldwide are highly sensitized to HLAs, characterized by elevated calculated panel-reactive antibody (cPRA) levels, with approximately 10–15% exhibiting cPRA values above 95% (1, 2). Within the Eurotransplant region, highly sensitized patients experience reduced transplantation rates due to inadequate compensation by the current Eurotransplant Kidney Allocation System for their limited access to transplantation (3). Consequently, patients with very high cPRA have minimal opportunities for identifying an HLA-compatible donor and frequently remain on dialysis for prolonged durations, resulting in increased morbidity, mortality, and financial costs compared to transplantation (4).

Probability analyses indicate that transplantation likelihood significantly declines as cPRA values approach 100%. A recent U.S. study identified 9,228 wait-listed patients with cPRA ≥99.5%, demonstrating that transplantation rates among these patients were critically influenced by their precise cPRA decile grouping. Indeed, patients in the two highest cPRA deciles exhibited notably lower transplantation rates (5). To address these challenges, potential strategies include precisely adjusting unacceptable antigens to reduce cPRA levels (6), expanding the donor pool through the acceptance of higher-risk donors, and implementing desensitization protocols for deceased and living donors, possibly combined with kidney-paired donation programs (7, 8).

Some highly sensitized KT candidates have potential living kidney donors; however, pre-existing donor-specific alloantibodies (DSA) often preclude transplantation in countries where kidney exchange programs (“swap” programs) are prohibited. The initial demonstration of a survival benefit associated with HLA-incompatible (HLAi) KT from living donors originated from the United States (9). In this cohort of 1,025 patients undergoing HLAi transplantation, significant survival benefits were observed compared to dialysis patients remaining on waiting lists, irrespective of subsequent transplantation. Importantly, this survival advantage persisted over eight years across varying levels of DSA. In contrast, a UK study by Mannook et al. found comparable survival rates between highly sensitized patients undergoing HLAi transplantation and dialysis patients awaiting compatible organs, many of whom were unlikely to be transplanted. Therefore, HLAi transplantation in the UK setting did not demonstrate a survival benefit, differing from outcomes reported by multicenter North American cohorts (10).

Several strategies exist for desensitizing highly HLA-sensitized patients (8). Primarily, these involve apheresis-based therapies designed to remove anti-HLA antibodies, frequently combined with B-cell depletion treatments such as rituximab and adjunctive immunosuppressive agents. Anti-interleukin-6 receptor monoclonal antibodies (e.g., tocilizumab) may further facilitate antibody clearance (11, 12). Conversely, polyclonal intravenous immunoglobulins (IVIg) alone have proven ineffective for HLA desensitization (13, 14), while combining rituximab and IVIg has demonstrated clinical efficacy (14). The role of imlifidase, a novel endopeptidase, remains under active investigation (15).

At our center, HLA desensitization protocols were initiated several years ago, incorporating semispecific immunoadsorption (IA)—an efficient technique for IgG antibody removal—in combination with rituximab, tacrolimus, mycophenolic acid (MPA), and corticosteroids. This therapeutic strategy has been successfully utilized in both living and deceased donor KT scenarios (16–18). The current study aimed to evaluate the influence of pretransplant crossmatch results on transplant outcomes among highly sensitized KT candidates undergoing IA-based desensitization protocols combined with rituximab, tacrolimus, MPA, and steroids.

## 2 Patients and methods

### 2.1 Patients

This single-center retrospective cohort study included patients enrolled in the HLAi- KT program initiated in May 2016. For recipients of deceased-donor kidneys, inclusion criteria were as follows: cPRA >85%, minimum of 3 years on the KT waiting list, absence of a suitable living donor, and willingness to undergo desensitization after providing informed consent. For recipients with living donor kidneys, desensitization was performed in case of positive CDC or FCM crossmatch. Prior to initiation of desensitization, all patients received vaccination against pneumococcal and meningococcal infections. During the entire desensitization protocol, antibiotic prophylaxis consisted of sulfamethoxazole-trimethoprim (400/80 mg every other day) and phenoxymethylpenicillin (1 million IU [M IU] bid). Desensitization involved semi-specific (removing only IgG from the sera but without any antigen-distinction) IA combined with hemodialysis (19), performed 5 times weekly for the initial 2 weeks, then tapered to 1–3 sessions per week based on reductions in anti-HLA mean fluorescence intensities (MFIs). HLA antigens corresponding to alloantibody MFIs below 3,000 were reclassified from forbidden to permissible antigens. Following desensitization, patients were allocated the first locally available deceased donor kidney for which they had no DSA with an MFI >3,000, effectively a negative virtual crossmatch.

In addition to IA, patients received two rituximab infusions (375 mg/m²), administered after the 5th and 10th IA sessions. Oral immunosuppressive therapy, initiated concurrently with the first IA session, consisted of tacrolimus (0.05 mg/kg bid, target trough levels 6–8 ng/mL), mycophenolate mofetil (MMF; 500 mg bid), and prednisone (0.25 mg/kg/day).

IA was performed on Comtech or Optia for the centrifugation step, followed by Adasord kit equipped with 2 columns (Globaffin), adsorbing alternately. The median volume was 6641 mL [5520 - 7523]. An optional filter (Monet) placed after centrifugation, was used to purify the plasma before adsorption. Substitution liquid was 100cc of Albumin 20% and/or Fresh Frozen Plasma. If plasma IgG levels fell below 2 g/L, 100 mg/kg of intra venous immunoglobulins were infused. If fibrinogen levels fell below 1 g/L, human fibrinogen was infused after the IA.

For recipients of living-donor kidneys, rituximab (375 mg/m²) was administered at 30 and 15 days before transplantation. Oral immunosuppressive therapy, including tacrolimus (0.05 mg/kg bid, target trough levels 6–8 ng/mL), MMF (500 mg bid), and prednisone (0.25 mg/kg/day), was initiated two weeks prior to transplantation. IA sessions were tailored according to the maximal pre-desensitization DSA MFI, with the objective of reducing maximal DSA MFI below 3,000 by the transplantation date. IA were continued as maintenance therapy until transplantation but discontinued at 4 months if no kidney was allocated. Anti-HLA monitoring was performed once a week during IA session and immediately before transplantation to evaluate the effectiveness of desensitization.

All desensitized patients received induction therapy at transplantation, consisting of anti-thymocyte globulin (ATG; 1 mg/kg/day for 5 consecutive days), and methylprednisolone pulses (500 mg on day 0, 250 mg on day 1, and 125 mg on day 2), followed by prednisone at 40 mg/day. Maintenance immunosuppressive treatment included tacrolimus (0.1 mg/kg bid, target trough levels 8–10 ng/mL) and MMF at 1 g bid for the initial 2 weeks, subsequently reduced to 500 mg bid. Prednisone was tapered to 10 mg/day starting on post-operative day (POD) 10, and further reduced to 5 mg/day beyond POD 90.

Post-transplantation prophylaxis involved sulfamethoxazole-trimethoprim (400/80 mg every other day for 6 months), phenoxymethylpenicillin (1 M IU bid for 12 months), and valganciclovir (900 mg/day, dosage adjusted based on estimated glomerular filtration rate) administered for 3–6 months according to cytomegalovirus (CMV) risk stratification.

The cohort of HLA-incompatible kidney transplant recipients was compared with a group of 160 highly sensitized patients (cPRA >85%) who underwent HLA-compatible (HLAc) kidney transplantation without desensitization. None of these patients had detectable pretransplant DSAs, and all had negative CDC crossmatches. Immunosuppressive management after transplantation in this HLAc cohort was similar, including induction therapy with anti-thymocyte globulin (ATG; 1 mg/kg/day for 5 consecutive days), methylprednisolone pulses (500 mg on day 0, 250 mg on day 1, and 125 mg on day 2), followed by prednisone at 40 mg/day. Maintenance immunosuppression consisted of tacrolimus (0.1 mg/kg bid, target trough levels 8–10 ng/mL) and MMF at 1 g bid for the first 2 weeks, subsequently reduced to 500 mg bid. Prednisone tapering was identical, reduced to 10 mg/day at POD 10 and 5 mg/day beyond POD 90.

The control group’s induction therapy consisted of ATG (1 mg/kg/day for 5 consecutive days), methylprednisolone pulses, and prednisone initiated at 40 mg/day. Maintenance immunosuppression included tacrolimus (0.1 mg/kg twice daily, target trough levels 8–10 ng/mL) and MMF (1 g twice daily for the first 2 weeks, then reduced to 500 mg twice daily). Post-transplant prophylaxis consisted of sulfamethoxazole-trimethoprim (400/80 mg every other day for 6 months) and valganciclovir (900 mg/day, dose-adjusted based on estimated glomerular filtration rate) for a duration of 3–6 months, determined by CMV risk stratification. Surveillance biopsies in this group were performed at month 3 post-transplantation, while for-cause biopsies were conducted whenever serum creatinine rose unexplainedly above 20% of baseline.

### 2.2 Surveillance

For recipients of HLAi KT, surveillance allograft biopsies were systematically performed at months 1, 3, and 12 post-transplantations. Additional kidney biopsies were conducted for-cause, triggered by an unexplained increase in serum creatinine exceeding 20% above baseline levels. All biopsies were evaluated by a single renal pathologist (DG) and scored according to the latest Banff classification criteria. Moreover, each biopsy was stained routinely for C4d and SV40 (20).

Post-transplant monitoring for anti-HLA alloantibodies was conducted on days 5, 15, and 30, and subsequently every month for the first 6 months. All rejection episodes required histological confirmation:

- For acute or chronic cellular rejection, patients received methylprednisolone pulses (10 mg/kg/day for 3 consecutive days), followed by a gradual prednisone taper. Steroid-resistant cases were treated with anti-thymocyte globulin (ATG; 1 mg/kg/day for 5 consecutive days).
- Acute antibody-mediated rejection (aABMR) treatment comprised methylprednisolone pulses (10 mg/kg/day for 3 consecutive days), plasmapheresis, rituximab, and intravenous immunoglobulins (IVIg). Patients exhibiting features of thrombotic microangiopathy were additionally treated with eculizumab.
- Treatment protocols for chronic active antibody-mediated rejection (caABMR) evolved over the study period. Prior to 2019, management consisted of two cycles of 3–4 double-filtration plasmapheresis sessions, each cycle concluding with rituximab infusion (375 mg/m²) (21). From 2019 onward, patients were treated with tocilizumab (8 mg/kg IV every 4 weeks indefinitely) (22).

In all groups, anti-HLA alloantibody monitoring occurred on post-transplant days 15, 30, then every two months during the initial 6 months, at month 12, and annually thereafter.

### 2.3 HLA typing, crossmatches, and anti-HLA alloantibody detection

#### 2.3.1 HLA-typing

Recipient and living donor HLA typing were performed independently on two separate samples using next-generation sequencing (NGS; Holotype HLA, Omixon, Budapest, Hungary) on an Illumina MiSeq platform, with analyses conducted via Twin software. Alternatively, typing was performed using sequence-specific oligonucleotide (SSO) technology (LIFECODES^®^ HLA typing kits, Immucor, Stamford, CT, USA) on a Luminex platform, with data interpreted using Match IT! DNA software according to the manufacturer’s guidelines.

HLA typing for deceased donors was initially conducted by real-time PCR (LinkSeq™, One Lambda™, ThermoFisher Scientific), with results subsequently confirmed through NGS (Holotype™, Omixon, Budapest, Hungary).

#### 2.3.2 Screening and identification of class I and II anti-HLA antibodies

Screening and identification of class I and II anti-HLA antibodies were performed using LIFECODES^®^ Lifescreen Deluxe and LIFECODES^®^ Single Antigen (LSA1 and LSA2) kits (Immucor, Stamford, CT, USA). Both assays were carried out on a Luminex platform and analyzed using Match IT! Antibody software, according to the manufacturer’s instructions.

In France, the cPRA equivalent, termed “TGI” (taux de greffons incompatibles), is expressed as a percentage. A TGI of 99%, for instance, indicates an extremely low probability of identifying a compatible HLA kidney donor. The TGI and cPRA values are virtually equivalent.

#### 2.3.3 Complement-dependent cytotoxicity crossmatch

Lymphocyte preparation differed according to donor type: lymphocytes from living donors were isolated from whole blood collected in ACD tubes, whereas lymphocytes from deceased donors were obtained by mechanical disruption and filtration of spleen tissue in RPMI medium. Cell concentrations (20–40 × 10^6^ lymphocytes/mL) were established using an automated hematology analyzer (ABX Micros ES 60, HORIBA Medical). Total lymphocytes (T+B) were isolated using a Ficoll density gradient, while specific T and B cell populations were purified using EasySep™ Direct HLA T and B Cell Isolation kits (STEMCELL Technologies), following manufacturer recommendations.

Recipient sera (historical and/or transplantation day samples), positive IgG controls (homemade), positive IgM controls (ALSM, One Lambda™), and negative controls (homemade) underwent treatment with dithiothreitol (DTT; GERBU™ 1008) at a 1:20 (v/v) ratio, incubated for 20 minutes at 37°C. Additionally, for patients treated with rituximab within the preceding 12 months, sera received further treatment with anti-rituximab antibody (clone 10C5, Abnova™) at a 1:5 (v/v) ratio.

CDC crossmatch assays involved adding 1 µL each of recipient serum (with or without DTT), negative controls, positive controls (IgM anti-lymphocyte control, with or without DTT), B lymphocyte monoclonal antibodies (1:20 dilution in human AB serum, ABSG, One Lambda™), and T lymphocyte monoclonal antibodies (1:100 dilution in human AB serum, ATSG, One Lambda™) into Terasaki plates. Subsequently, 1 µL of lymphocyte suspension (2–4 × 10^6^ lymphocytes/mL; total T+B, isolated T, or isolated B cells) was added per well, incubated for 45 minutes at room temperature, followed by addition of 5 µL rabbit complement (CL3111, Cedarlane™) and incubation for 90 minutes. Reactions were terminated with 2 µL Fluoroquench™ (FQAE100, One Lambda™). Crossmatch results were interpreted under an inverted fluorescence microscope (Leitz Diavert) according to the American Society for Histocompatibility and Immunogenetics (ASHI) scoring criteria.

#### 2.3.4 Flow-cytometry crossmatch

Lymphocytes from living and deceased donors were isolated either from whole blood collected in ACD tubes or through mechanical disruption and filtration of spleen samples in RPMI medium, followed by Ficoll density gradient separation. Cell concentrations were adjusted to 10 × 10^6^ cells/mL using an automated hematology analyzer (ABX Micros ES 60, HORIBA Medical).

For patients treated with rituximab in the prior 12 months, sera underwent additional treatment with anti-rituximab antibody (clone 10C5, Abnova™) at a 1:5 (v/v) ratio.

Fifty µL of lymphocyte suspension (10 × 10^6^ cells/mL) was combined with 50 µL of recipient serum, IgG-positive, and IgG-negative controls (homemade), and incubated at room temperature for 30 minutes. Cells were subsequently washed twice with PBS (1×), followed by centrifugation at 1800g for 5 minutes. After washing, cells were incubated in the dark for 20 minutes with 3 µL anti-CD3-PC7 (clone UCHT1, Beckman-Coulter™), 3 µL anti-CD19-APC (clone J3-119, Beckman-Coulter™), 1 µL anti-CD45-BV421 (clone HI30, BD Biosciences™), and 100 µL goat F(ab’)2 anti-human IgG-FITC antibody (1:100 dilution in PBS; Jackson™). Post-incubation, cells underwent an additional PBS wash at 1800g for 5 minutes, and pellets were resuspended in 250 µL PBS.

Flow cytometric acquisition was performed using a BD FACS Canto II instrument (BD Biosciences™), and analyses were conducted using FACSDiva™ software (version 6.1.3).

### 2.4 Data collection

Data for this study were retrospectively extracted from electronic medical records. All medical data were collected from our database [CNIL (French National committee for data protection) approval number 1987785v0]. These included demographic characteristics such as patient age and sex; immunological parameters such as HLA typing, anti-HLA alloantibodies and their MFIs, and CDC and FCM crossmatch results; and biological parameters, including hematological and biochemical data. Infectious and non-infectious post-transplant complications and histological findings from kidney allograft biopsies were also systematically documented.

Annual follow-up beyond the first post-transplant year included vital status (alive or deceased) and graft function status (functioning or non-functioning). For functioning grafts, serum creatinine, estimated glomerular filtration rate (eGFR), microalbuminuria (mg/L), presence or absence of DSA, and evidence of rejection were recorded.

### 2.5 Statistical analysis

Quantitative variables with a normal distribution were presented as mean ± standard deviation (SD), while those with a non-normal distribution were presented as median with interquartile ranges (IQR). Qualitative variables were reported as counts and percentages.

For statistical comparisons among multiple groups, the Kruskal-Wallis test was employed. For comparisons between two groups, the unpaired Mann-Whitney U test was used. Categorical data were compared using the chi-squared test.

Patient survival and death-censored graft survival curves of each group was estimated using Kaplan-Meier method and compared using the log rank test. Statistical significance was set at p < 0.05. All statistical analyses and figures were performed using RStudio software (Version 2024.12.0 + 46).

## 3 Results

### 3.1 Patient demographics and baseline characteristics

All patients included in the study had a cPRA greater than 85% either pre-transplant or pre-desensitization. This group was compared to a historical cohort comprising 160 highly sensitized kidney transplant recipients (cPRA >85%), who received HLAc KT between February 1, 2009, and June 1, 2022. The historical control group was assessed retrospectively and had no detectable DSA at the time of transplantation and had negative CDC crossmatch. In contrast, 49 patients received HLA-incompatible kidney transplants (HLAi KT), of whom 23 required pre-transplant desensitization due to a positive CDC or flow cytometry (FCM) crossmatch. Demographic and clinical characteristics are detailed in **Table 1**. Pre-transplant comparisons revealed significantly more living donors (p<0.001) and greater HLA A/B/DR/DQ mismatches in the HLAi group (p=0.027). Among the 49 patients that underwent HLAi KT, 25 (51%) received kidneys from deceased donors and 28 (57.1%) were male.

**Table 1:** Demographics and post-transplant data of HLA sensitized patients with HLA compatible (HLAc) or HLA incompatible (HLAi) kidney transplantation.

Characteristics	HLAc (n=160)	HLAi (n=49)	Total (n=209)	P-value
Age (years)	54 ± 13.2	51 ± 12	53.3 ± 13.2	0.16
cPRA (%)	93.7 ± 4.7	73 ± 37	88.8 ± 20.3	0.51
Dialysis vintage (years)	5.7 ± 5.9	8.3 ± 9.3	6.3 ± 6.9	0.18
Gender (M/F; %)	40.6 / 59.4	42.9 / 57.1	41.1 / 58.9	0.78
Donor Type (%) Living Deceased	3.1 96.9	49 51	13.9 86.1	< 0.001
MM numbers	5.7 ± 1.2	5.8 ± 2	5.7 ± 1.4	0.027
ATG (total dose; mg)	308 ± 87	338 ± 108	315 ± 93	0.104
DGF (%)	11	14.6	11.9	0.5
Patient/graft survival at 1y (%)	95/93.75	100/93.8	96.6/93.7	ns
BPAR 1st year (Yes) (%)	3.1	24.5	8.1	< 0.001

cPRA, calculated Panel Reactive Alloantibodies; M, male; F, female; MM HLA antigen (A, B, DR, DQ) mismatches; ATG, antithymocyte globulins; DGF, delayed graft function; 1y, 1 year post-transplantation; BPAR, biopsy proven acute rejection within the first year post-transplantation.

Retrospectively, 23 had at least a positive CDC or FCM crossmatches pre-transplant. All patients with a positive crossmatch had at least 1 DSA. The mean number of DSA per patient was 2.7. The sum of all DSA MFI (including class I and II DSAs) before desensitization was 20,716. Twenty-six patients did not have any positive crossmatch: 8 patients had a living donation with a positive DSA in Luminex whose MFI was not high enough to result in a positive CDC or FCM. 18 patients received a kidney from a deceased donor after a period of desensitization with a low pre-existing DSA, because of the allocation system algorithm. Within the HLAi group, crossmatch-negative patients were older and had fewer living donors compared to crossmatch-positive patients.

**Table 2** compares groups according to the crossmatch results. Of the 23 desensitized patients with at least one positive crossmatch (CDC or FCM), 7 (30.4%) received kidneys from deceased donors (more frequently in the negative crossmatch group: 68% vs. 30.4%, p=0.009). All, but one patient, were on chronic hemodialysis (median duration: 10.2 years; range: 5.3 – 11.8). Mean patient age was 47.2 ± 12.4 years, and 12 (52.2%) were female, comparable between both groups (p=0.08 and p=0.4 respectively). Pre-transplant hypertension was present in seven patients, and two had type 2 diabetes. Retransplantation was performed in 12 patients (52.1%). High HLA A/B/DR/DQ mismatches (>4) occurred in 19 patients (82.6%), with a median TGI score of 98% (range: 95.5–99%) (More data on the 23 HLAi KT with a positive crossmatch are available in **Supplementary Table 1**). All received induction therapy with ATG (median dose: 360 mg; range: 250–387.5 mg). Delayed graft function (dialysis beyond day 7 post-transplant) occurred in five patients (22.7%), more frequently in the positive crossmatch group (22.7% vs 4%, p=0.05). Within the first post-transplant year, biopsy-proven acute rejection (BPAR) occurred in eight patients (34.8%), including both cellular and antibody-mediated rejections.

**Table 2:** Comparison of CDC or FCM (+) and crossmatch negative patients (demographics and post-transplant data).

Characteristics	CDC or FCM (+) (n=23)	CDC and FCM (–) (n=26)	P-value
Age (years)	47.2 ± 12.4	53.7 ± 12.4	0.083
Donor type (n; %):			
*Living* *Deceased*	16 (69.6) 7 (30.4)	8 (32) 18 (68)	0.009
Dialysis vintage (years)	9.4 ± 10.2	8.5 ± 7.9	0.11
Gender (M/F) (%)	47.8 / 52.2	36 / 64	0.40
MM numbers	6.0 ± 2.0	5.6 ± 2.0	0.26
ATG total dose (mg)	332 ± 112	343 ± 107	0.96
DGF (%)	5 (22.7)	1(4)	0.055
Patient/graft survival at 1y (%)	100/95.6	100/92.3	ns
BPAR 1rst year (Yes) (%)	8 (34.8)	4(16)	0.133

CDC, lymphocytotoxic crossmatch test; FCM, flow cytometry crossmatch test; M, male; F, female; MM HLA mismatching (HLA A, B, DR, DQ); ATG, antithymocyte globulins; DGF, delayed graft function; 1y, 1-year post-transplantation; BPAR, biopsy proven acute rejection within the first-year post-transplantation.

Finally, before desensitization, among the 23 patients with at least a positive crossmatch (CDC and/or FCM), 15 patients (65.2%) had a positive CDC crossmatch, all of which became negative at transplantation. Flow cytometry identified 18 patients (78.2%) with positive crossmatches, including three additional patients with initially negative CDC results.

### 3.2 Comparison according to CDC and FCM results

Comparison between highly sensitized HLAc and HLAi KT based on CDC crossmatch results is shown in **Table 3**. Significantly more deceased donor transplants were in the control group (96.2% vs. 50.0% and 73.3% in the HLAi negative and positive CDC respectively, p < 0.001). High HLA A/B/DR/DQ mismatches (>4) were also more frequent in the control group (91.2% vs. 87.5% and 80.0% in the HLAi negative and positive CDC respectively, p = 0.002). Induction therapy, ATG doses, and tacrolimus levels were similar across groups. Delayed graft function, peritransplant bleeding, and infectious complications rates were similar; however, peritransplant collections were higher in the desensitized group with negative CDC (25% vs. 9.1% in the HLAc group and vs. 6.6% in the HLAi with positive CDC, p = 0.03). The BPAR rate within one year was significantly higher in the CDC-positive group (26.7%) compared to CDC-negative (12.5%) and control groups (3.1%, p < 0.001).

**Table 3:** Baseline characteristics of HLA compatible and HLA incompatible KTx recipients according to CDC crossmatch results.

Characteristics	HLAc (N=160)	HLAi/ Neg CDC (N=8)	HLAi/Pos CDC (N=15)	Total (N=183)	p value
Donor					< 0.001
Deceased	154 (96.2%)	4 (50.0%)	11 (73.3%)	169 (92.3%)	
Living	6 (3.8%)	4 (50.0%)	4 (26.7%)	14 (7.7%)	
Recipient gender					0.640
Female (%)	94 (58.8%)	5 (62.5%)	7 (46.7%)	106 (57.9%)	
Male (%)	66 (41.2%)	3 (37.5%)	8 (53.3%)	77 (42.1%)	
Transplant rank					0.788
1	50 (31.2%)	4 (50.0%)	7 (46.7%)	61 (33.3%)	
2	92 (57.5%)	3 (37.5%)	6 (40.0%)	101 (55.2%)	
3	17 (10.6%)	1 (12.5%)	2 (13.3%)	20 (10.9%)	
4	1 (0.6%)	0 (0.0%)	0 (0.0%)	1 (0.5%)	
Pretransplant dialysis					0.773
No (%)	8 (5.0%)	0 (0.0%)	1 (6.7%)	9 (4.9%)	
Yes (%)	152 (95.0%)	8 (100.0%)	14 (93.3%)	174 (95.1%)	
Dialysis vintage (years)					0.094
Mean (SD)	5.8 (5.9)	6.0 (4.5)	9.6 (9.4)	6.1 (6.3)	
Median (Q1, Q3)	4.0 (2.2, 7.2)	6.6 (2.7, 9.3)	6.9 (4.9, 8.8)	4.4 (2.2, 7.8)	
Missing data	8	0	0	8	
BMI (kg/m2)					0.759
Mean (SD)	23.8 (5.0)	25.2 (5.9)	23.6 (5.2)	23.9 (5.0)	
Median (Q1, Q3)	23.1 (20.1, 26.4)	25.4 (21.9, 29.5)	22.1 (20.3, 27.8)	23.1 (20.1, 26.7)	
Recipient (age years)					0.085
Mean (SD)	53.7 (13.3)	47.3 (12.3)	46.7 (13.4)	52.9 (13.4)	
Median (Q1, Q3)	54.6 (42.7, 65.0)	44.2 (41.3, 58.2)	47.3 (37.8, 53.5)	53.2 (42.1, 64.1)	
Cold ischemia time (minutes)					0.490
Mean (SD)	1199.9 (720.5)	975.5 (754.7)	1117.0 (769.0)	1183.2 (723.5)	
Median (Q1, Q3)	1155.0 (770.0, 1657.5)	882.0 (626.0, 1046.0)	931.0 (592.0, 1752.5)	1142.5 (714.0, 1631.2)	
Donor age (years)					0.794
Mean (SD)	55.7 (14.8)	58.8 (7.5)	55.3 (10.4)	55.8 (14.2)	
Median (Q1, Q3)	57.5 (47.0, 66.0)	56.0 (52.8, 65.2)	56.0 (49.0, 60.0)	57.0 (47.5, 66.0)	
Donor gender					0.027
Female (%)	66 (41.2%)	7 (87.5%)	5 (33.3%)	78 (42.6%)	
Male (%)	94 (58.8%)	1 (12.5%)	10 (66.7%)	105 (57.4%)	
HLA ABDRDQ mismatches					0.002
1	0 (0.0%)	0 (0.0%)	1 (6.7%)	1 (0.5%)	
2	1 (0.6%)	1 (12.5%)	1 (6.7%)	3 (1.6%)	
3	5 (3.1%)	0 (0.0%)	0 (0.0%)	5 (2.7%)	
4	8 (5.0%)	0 (0.0%)	1 (6.7%)	9 (4.9%)	
5	61 (38.1%)	2 (25.0%)	1 (6.7%)	64 (35.0%)	
6	49 (30.6%)	2 (25.0%)	3 (20.0%)	54 (29.5%)	
7	21 (13.1%)	3 (37.5%)	5 (33.3%)	29 (15.8%)	
8	15 (9.4%)	0 (0.0%)	3 (20.0%)	18 (9.8%)	
Desensitization setting					< 0.001
ABOi HLAi	0 (0.0%)	2 (25.0%)	0 (0.0%)	2 (1.1%)	
HLAi	3 (1.9%)	6 (75.0%)	15 (100.0%)	24 (13.1%)	
None	157 (98.1%)	0 (0.0%)	0 (0.0%)	157 (85.8%)	
TGI (%)					0.026
Mean (SD)	93.7 (4.7)	94.6 (5.0)	96.7 (4.0)	94.0 (4.7)	
Median (Q1, Q3)	95.0 (89.8, 98.0)	95.5 (93.2, 99.0)	98.0 (97.0, 99.0)	95.0 (90.0, 98.0)	
Induction therapy					0.978
ATGAM	1 (0.6%)	0 (0.0%)	0 (0.0%)	1 (0.6%)	
Basiliximab	2 (1.3%)	0 (0.0%)	0 (0.0%)	2 (1.1%)	
ATG	151 (98.1%)	8 (100.0%)	15 (100.0%)	174 (98.3%)	
Missing data	6	0	0	6	
ATG total dose (mg)					0.524
Mean (SD)	349.2 (509.8)	328.1 (68.9)	331.7 (122.8)	346.8 (476.4)	
Median (Q1, Q3)	265.0 (250.0, 375.0)	350.0 (306.2, 375.0)	375.0 (250.0, 400.0)	280.0 (250.0, 375.0)	
Missing data	8	0	0	8	
Dialysis by D7 post-transplant					0.314
No (%)	130 (88.4%)	8 (100.0%)	11 (78.6%)	149 (88.2%)	
Yes (%)	17 (11.6%)	0 (0.0%)	3 (21.4%)	20 (11.8%)	
Missing data (n)	13	0	0	13	
Tacrolimus trough level (ng/mL)					0.548
Mean (SD)	7.3 (3.5)	7.3 (1.9)	6.4 (4.0)	7.2 (3.5)	
Median (Q1, Q3)	7.0 (5.0, 8.0)	6.8 (6.0, 8.5)	6.0 (4.0, 8.5)	7.0 (5.0, 8.0)	
Missing data (n)	8	0	0	8	
Bleeding complications					0.387
No (%)	148 (96.1%)	8 (100.0%)	13 (86.7%)	169 (95.5%)	
Yes (%)	6 (3.9%)	0 (0.0%)	2 (13.3%)	7 (4.5%)	
Missing data (n)	6	0	0	6	
Peritransplant collection					0.031
Yes (%)	14 (9.1%)	2 (25.0%)	1 (6.6%)	17 (9.6%)	
No (%)	140 (90.9%)	6 (75.0%)	14 (93.4%)	160 (90.4%)	
Missing data (n)	6	0	0	6	
Infectious complications					0.797
No (%)	125 (81.2%)	7 (87.5%)	13 (86.7%)	145 (81.9%)	
Yes (%)	29 (18.8%)	1 (12.5%)	2 (13.3%)	32 (18.1%)	
Missing data (n)	6	0	0	6	
Patient / graft survival A at 1-year (%)					
BPAR at M12 post-transplant					< 0.001
No (%)	155 (96.9%)	7 (87.5%)	11 (73.3%)	173 (94.5%)	
Yes (%)	5 (3.1%)	1 (12.5%)	4 (26.7%)	10 (5.5%)	

KTx, kidney transplant; HLA, human leukocyte antigen; HLAc, HLA compatible; HLAi, HLA incompatible; pos, positive, neg, negative; CDC, complement-dependent cytotoxicity; BMI, body mass index; D, day; TGI, taux de greffons incompatibles; ATG, antithymocyte globulins; ABOi, ABO incompatible; BPAR, biopsy-proven acute rejection; M, month.

**Supplementary Table 2** compares groups according to FCM results. HLA A/B/DR/DQ mismatches were significantly higher in the HLAc group (87.5% vs. 77.7%, p = 0.004). Deceased donor transplants were more frequent, and TGI scores lower, in the HLAc group. At 12 months post-transplant, BPAR was significantly higher in the FCM-positive group (27.7%) versus the HLA-compatible group (3.1%, p < 0.001).

### 3.3 Allograft losses and patients deaths

**Figure 1:**
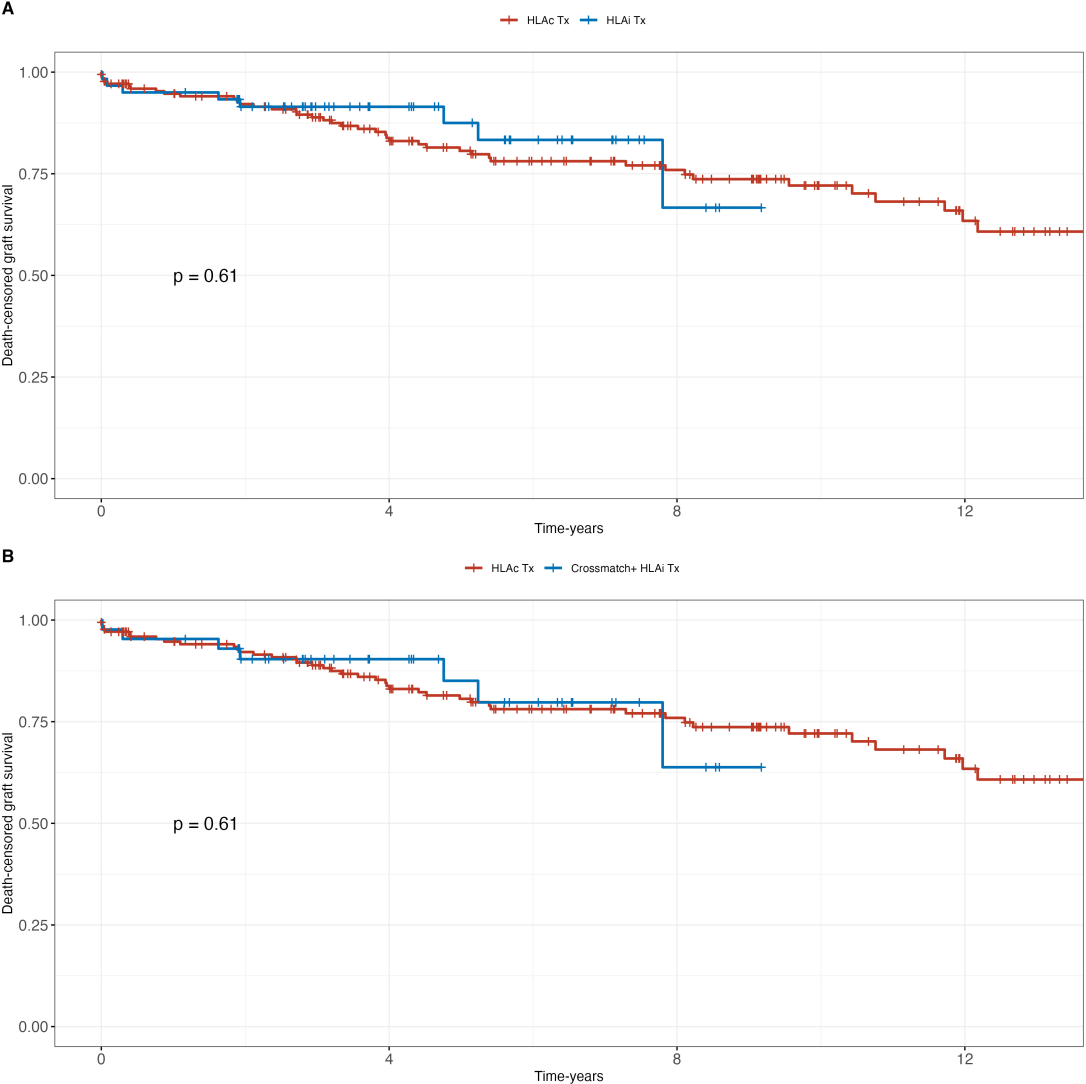
Patient survival; **(A)** HLA incompatible (HLAi) kidney transplant patients (KTx) vs. HLA compatible (HLAc) KTx; **(B)** crossmatch positive patients (HLAi) vs. HLAc KTx.

**Figure 2:**
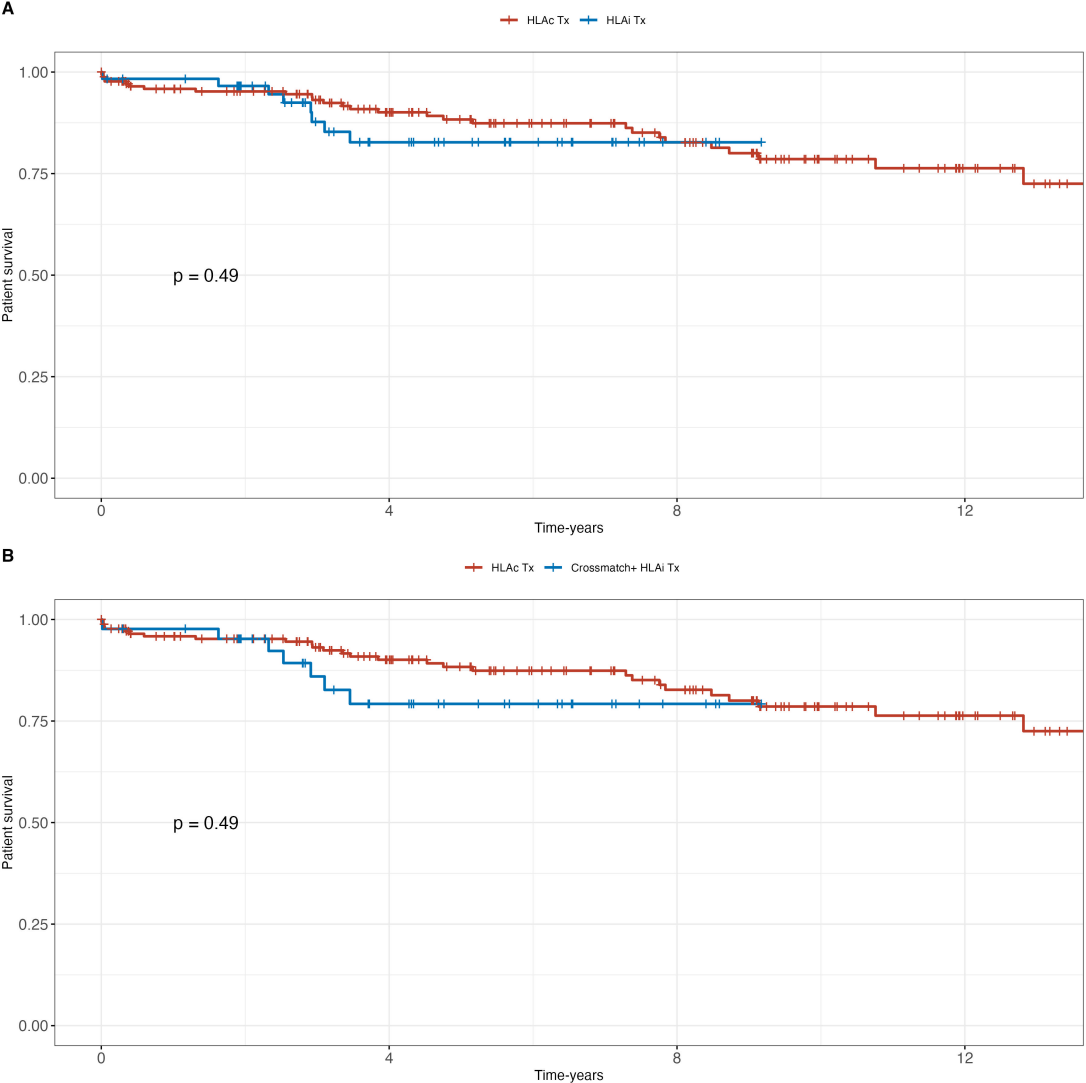
Death-censored graft survival (DCGS): **(A)** HLA incompatible (HLAi) kidney transplant patients (KTx) vs. HLA compatible (HLAc) KTx; **(B)** crossmatch positive patients (HLAi) vs. HLAc KTx.

No significant differences were observed in patient and death-censored graft survival between HLAc and HLAi patients or between HLAc and CDC-positive HLAi patients (**Figures 1A, B**, **2A, B**). Graft survival probability at 1-year was 95% [95%IC 93 – 98] and 97 [95%IC 93 – 100] in the HLAc and CDC-positive HLAi group (p=ns). Median follow-up durations post-desensitization/transplantation were 7 years (range: 2–8 years) in CDC-positive and 6 years (range: 3–8 years) in CDC-negative patients.

Median time to graft loss was 3.8 years (1.8–7.8), 4.1 years (2.3–6.1), and 1.9 (0.9–4.9) years in the HLAc, CDC-negative HLAi, and CDC-negative HLAi groups respectively. All graft losses occurred in patients who received their graft from a deceased donor except 1 patient in the group CDC-positive HLAi who lost his living donor graft after 7.8 years. Median time to patient death was 2.7 (2.1–2.9) years, 2.5 (1.9–3.0) years, and 2.9 (2.9–2.9) years in the HLAc, CDC-positive HLAi, and CDC-negative HLAi group respectively (p=0.45). One patient who received a living donor kidney died in the CDC-positive HLAi group at 3.1 years and 1 patient in the CDC-negative HLAi at 2.9 years. No patients who received a graft from a living donor died in the HLAc group. Three patients died in the desensitized group: one with excellent renal function experienced sudden death, while two others died after caABMR and subsequent dialysis resumption.

### 3.4 Rejection

In the CDC-positive group (n=15), three patients developed caABMR with graft loss; one subsequently died on dialysis. Additionally, one patient died with a functioning graft. In the CDC-negative group (n=8), one patient experienced caABMR leading to graft loss and death after returning to dialysis.

### 3.5 Renal function

At the last follow-up, median serum creatinine was 120 µmol/L (range: 82–360) with eGFR of 44 mL/min (range: 17–77) in the CDC-positive group, and 134 µmol/L (range: 79–164) with eGFR of 47 mL/min (range: 37–85) in the CDC-negative group. Microalbuminuria was undetectable in six CDC-positive patients, while five had median albuminuria of 200 mg/L (range: 84–965). Among CDC-negative patients, six had undetectable albuminuria; two had values of 610 mg/L and 1100 mg/L, respectively.

### 3.6 Infectious complications

Finally, the rate of 1-year infectious complications was similar between HLAc and HLAi (with or without positive CDC crossmatch) patients (**Table 3**).

## 4 Discussion

Our study demonstrates that IA-based desensitization enables successful kidney transplantation in highly sensitized patients (cPRA >85%), even those with pretransplant positive crossmatches (CDC and/or FCM). The most notable finding was the comparable long-term patient and graft survival between desensitized and HLA-compatible (HLAc) groups (7-year graft survival: 93.8% vs. 93.75%, p=0.16).

It is noteworthy that in Australia, the implementation of cPRA resulted in a significant rise in the proportion of patients categorized as highly sensitized, using a threshold of PRA/cPRA ≥80% (2). This variation in definitions across regions underscores the need for localized criteria; hence, we adopted a cPRA >85% threshold to define highly sensitized patients in our cohort.

IA, when coupled with concurrent hemodialysis, optimizes patient management by reducing time spent in treatment sessions. Additionally, our previous studies indicated that adjunctive therapy with tocilizumab effectively managed antibody rebound in patients with high baseline MFIs.

However, desensitized patients experienced higher rates of antibody-mediated rejection (ABMR; 26.7% in CDC-positive vs. 3.1% in HLAc), underscoring the need for vigilant post-transplant monitoring. The discordance between Luminex-detected DSAs and crossmatch results (e.g., FCM negativity in patients with low-MFI DSAs) highlights the importance of integrating multiple assays to assess immunological risk.

Despite higher rejection rates, infectious complications and delayed graft function (DGF) did not differ significantly between desensitized and HLAc groups, aligning with prior studies (23). Notably, peritransplant collections were more frequent in CDC-negative desensitized patients, possibly reflecting protocol-driven fluid management in this subgroup. These findings support IA’s advantage over plasmapheresis, as it selectively removes IgG without depleting coagulation factors (24).

Another therapeutic agent, imlifidase—an IgG-cleaving enzyme—has recently received conditional approval by the EMA for highly sensitized patients (cPRA ≥95%). However, given immediately before transplantation without prior desensitization, nearly half of the recipients experience significant antibody rebound between days 5 to 21 post-transplant, frequently resulting in severe antibody-mediated rejection (15).

Safety remains a critical consideration in desensitization protocols. Studies from the US and UK demonstrated equivalent or improved survival among HLA-desensitized living-donor kidney transplant recipients compared to highly sensitized patients remaining on waiting lists (9, 10). Such data reassure clinicians about the safety and viability of desensitization approaches.

Our findings confirm that pretransplant IA combined with standard immunosuppression is safe and efficient and demonstrates comparable outcomes to those observed in highly sensitized patients undergoing HLAc transplantation in our center. We have chosen IA instead of DFPP or plasmapheresis (PP) because IA is more efficient at removing anti-HLA alloantibodies. Indeed, Jambon et al. have assessed the initial MFI of 1416 positive beads with MFIs obtained after 7 to 8 apheresis sessions, either PP or IA in the setting of pretransplant HLA desensitization. They have shown that MFI reduction after extended apheresis protocol was stronger with IA [87% (61%-100%)] than with PP [73% (22%-100%)] (P <.001). In addition, 59% of the beads had a final MFI < 2000 with IA, whereas only 38% with PP (P <.001) (23). Likewise, In ANCA-associated vasculitis Liu et al. have shown that IA was effective in the removal of MPO-ANCA and IgG, and showed superior over PP in the clearance of MPO-ANCA within 1 month after treatment; in addition, after a median follow-up of 14.5 months, IA therapy showed an advantage in reducing mortality over PP (25). In addition, IA is more practical as compared to PP because it does not require replacement fluid, and safer as compared to DFPP or PP because it removes fewer clotting factors (24, 26).

When it comes to HLA highly sensitized kidney transplant recipients the major issue is the long-term results. Eurotransplant has developed the acceptable mismatch (AM) program, i.e., acceptable antigens are defined as HLA antigens to which the patient has never made antibodies. This strategy aims at the prediction of a negative cross match (27). Since the start of the program almost 2000 patients participated and more than 1000 patients were transplanted with excellent transplant outcome, comparable to that of non-immunized transplant recipients within Eurotransplant (27). Indeed, patients that were transplanted through the AM program had a similar rejection incidence and long-term graft survival rates identical to non-sensitized patients transplanted through regular allocation (28). However, a subset of patients included in the AM program does not receive an organ offer within a reasonable time frame. As these are often patients with a rare HLA phenotype in comparison to the Eurotransplant donor population, extension of the donor pool for these specific patients through further European collaboration would significantly increase their chances of being transplanted. Indeed, this was recently demonstrated in a study encompassing various European bodies such as Eurotransplant, UK National Health Service Blood and Transplant, Barcelona, Prague and Athens focused on long waiting highly sensitized patients: results from simulations using newly developed tool showed that 195 (27%) of the 724 long waiting highly sensitized patients registered at each partner organization have increased chances of transplant in a different European donor pool (29).

However, for those patients that will not benefit from such strategy, desensitization is the ultimate solution. Indeed, in the last years very few studies have addressed the long-term results of crossmatch positive desensitized KT recipients. In the setting of living donors Yilmaz et al. have try to desensitized with plasmapheresis/rituximab/IVIG 49 kidney transplant candidates with positive crossmatches; however, only 16 undergone successful kidney transplantation; AMR and acute T cell-mediated rejection rates were 18.8% and 6.3%, respectively; graft survival rates at the first, third, and fifth years post-transplantation were 93.8%, 85.2%, and 85.2%, respectively (30). Kahwaji et al. reported on 66 sensitized KT candidates of whom 53 had a positive crossmatch including 48 with a positive FCM. They were desensitized with IVG plus rituximab (31). Results were compared to an historical cohort of 111 low-risk patients. At 6 years post-transplantation patient and graft survival were similar across the two groups, as well as allograft rejection rates even though there was a trend for higher rate in the desensitized group (30%) as compared to the low-risk group (23%). Finally, Kim et al. retrospectively analyzed living donor KT recipients some of whom having CDC +/FCM + or CDC-/FCM+ crossmatches and for which pretransplant desensitization relied on plasmapheresis, IVIG, rituximab with/without bortezomib and compared the long-term results to those having CDC-FCM-positive crossmatches (32). They found that death-censored graft survival and patient survival were not different among the three groups, even though crossmatch positive groups had significantly more BPAR and a worst renal function.

In this series of HLAi KT recipients with a positive CDC crossmatch before desensitization most of them were recipients of a deceased donor. In Europe there are very few reports on such a population apart from those who are desensitized with Imlifidase (33). The group of Vienna has reported on a series of 101 DSA+ deceased-donor KT recipients who were subjected to IA-based desensitization; treatment included a single pre-transplant IA session, followed by anti-lymphocyte antibody and serial post-transplant IA. In 27 cases, a positive CDC crossmatch was rendered negative by the pretransplant IA session (34). The three-year death-censored graft survival in DSA+ patients was significantly worse than in 513 DSA- recipients transplanted during the same period (79 versus 88%, P = 0.008). In addition, a positive baseline CDC crossmatch showed only a trend towards higher ABMR rates (41 versus 30% in CDC crossmatch negative recipients). Our series is the first one to demonstrate a different option, i.e., pretransplant IA plus conventional immunosuppression for desensitization with CDC positive crossmatches to start with, ending up with very good long-term results. Finally, the rate of 1-year infectious complications was very low (13%), i.e., similar to that observed in highly sensitized patients receiving an HLAc kidney transplant. This is at odds to the results reported by Bureau et al. on a small series of 15 highly sanitized patients desensitized pretransplant with IA-based therapy. They found that eight patients (53.3%) developed severe infections, including two fatal outcomes (30). There is no obvious explanation for this huge discrepancy.

The limitations of our study are that there were quite a few numbers of CDC positive desensitized KT recipients, and that it was a single center study. Finally, a cost-analysis is necessary as compared to a strategy based on Imlifidase desensitization. Our study has limitations: its single-center design, small cohort (*n* = 23 CDC-positive patients), and lack of cost-analysis versus newer therapies (e.g., imlifidase). Additionally, the control group was retrospectively enrolled, which may introduce selection bias. Multicenter studies with standardized protocols are needed to validate long-term outcomes and optimize desensitization strategies.

## 5 Conclusion

This study confirms that pre-transplant desensitization using semi-specific IA effectively enables successful KT in highly sensitized patients with positive crossmatches. Despite a higher incidence of ABMR, patient and graft survival rates were comparable to HLAc KT, with no increase in infectious complications. IA proved more efficient than plasmapheresis, offering better HLA antibody clearance with fewer coagulation factor losses. While promising, these findings require validation in larger multicenter studies to further assess long-term outcomes and cost-effectiveness.

## Data Availability

The datasets presented in this study can be found in online repositories. The names of the repository/repositories and accession number(s) can be found in the article/**Supplementary Material**.
