# Supplementary material for: Kidney transplant outcomes in HLA desensitized patients with pretransplant CDC and/or FCM positive crossmatches
Source: Front Immunol. 2025 Jun 23;16:1612462. doi: 10.3389/fimmu.2025.1612462 (PMC12229852; doi:10.3389/fimmu.2025.1612462)
Supplement: Supplementary file 1 [file Table1.docx]

**Supplementary table 1:** Demographic data of the 23 HLA incompatible KTx with pretransplant positive crossmatches.

| **Desensitized patients** | N=23 |
| --- | --- |
| **Donor type** |  |
| Deceased | 16 (69.6%) |
| Living | 7 (30.4%) |
| **Recipient gender** |  |
| Female (%) | 12 (52.2%) |
| Male (%) | 11 (47.8%) |
| **Transplant rank** |  |
| 1 | 11 (47.9%) |
| 2 | 9 (39.1%) |
| 3 | 3 (13.0%) |
| **Dialysis vintage (years)** |  |
| Mean (SD) | 9.4 (10.2) |
| Median (Q1, Q3) | 10.2 (5.3, 11.8) |
| **Recipient age (years)** |  |
| Mean (SD) | 46.9 (12.7) |
| Median (Q1, Q3) | 45.0 (39.2, 56.3) |
| **Donor age (years)** |  |
| Mean (SD) | 56.5 (9.5) |
| Median (Q1, Q3) | 56.0 (51.0, 62.5) |
| **Donor gender** |  |
| F | 12 (52.2%) |
| M | 11 (47.8%) |
| **History of diabetes** |  |
| No (%) | 21 (91.3%) |
| Yes (%) | 2 (8.7%) |
| **ATG total dose (mg)** |  |
| Mean (SD) | 332 (112) |
| Median (Q1, Q3) | 360.0 (250.0, 387.5) |
| **Post-transplant dialysis at D7** |  |
| No (%) | 18 (78.2%) |
| Yes (%) | 5 (22.7%) |
|  |  |
| **Tacrolimus trough level (ng/mL) over the 1st year post-KTx** |  |
| Mean (SD) | 6.7 (3.4) |
| Median (Q1, Q3) | 6.5 (4.5, 8.5) |
| **Peritransplant bleeding** |  |
| No (%) | 21 (91.3%) |
| Yes (%) | 2 (8.7%) |
| **Infectious complications** |  |
| No (%) | 20 (87.0%) |
| Yes (%) | 3 (13.0%) |
| **BPAR at 12 months post-transplant** |  |
| No (%) | 15 (78.3%) |
| Yes (%) | 8 (34.8%) |
| **Desensitization setting** |  |
| ABOi HLAi | 2 (8.7%) |
| HLAi | 21 (91.3%) |
| **TGI (%)** |  |
| Mean (SD) | 96.0 (4.4) |
| Median (Q1, Q3) | 98.0 (95.5, 99.0) |
| **DSA** |  |
| N | 1 (4.5%) |
| O | 21 (95.5%) |
| Missing data (n) | 1 |
| **Class I DSA** |  |
| N | 4 (18.2%) |
| O | 18 (81.8%) |
| Missing data (n) | 1 |
| **Class II DSA** |  |
| N | 6 (27.3%) |
| O | 16 (72.7%) |
| Missing data (n) | 1 |
| **Total class I DSA** |  |
| Mean (SD) | 2.0 (1.4) |
| Median (Q1, Q3) | 2.0 (1.0, 2.8) |
| Missing data (n) | 1 |
| **Total class II DSA** |  |
| Mean (SD) | 1.5 (1.5) |
| Median (Q1, Q3) | 1.0 (0.2, 2.0) |
| Missing data (n) | 1 |
| **Total DSA MFI** |  |
| Mean (SD) | 32913.4 (25511.1) |
| Median (Q1, Q3) | 27181.0 (20391.0, 37131.0) |
| Missing data (n) | 2 |

*Abbreviations*: KTx, kidney transplantation; F, female; M, male; HLAi, HLA incompatible KTx; ABOi; ABO incompatible KTx; BPAR, biopsy-proven acute rejection; TGI, rate of deceased-donor incompatible transplants over the last 5 years in France; D, day; DSA, donor-specific alloantibody; Y, yes; N, no; MFI, mean fluorescence intensity.

**Supplementary table 2:** Baseline characteristics of HLA compatible and HLA incompatible KTx recipients according to FCM crossmatch results

|  | **HLAc (N=160)** | **HLAi/ Neg FCM (N=5)** | **HLAi/Pos FCM (N=18)** | **Total (N=183)** | **p value** |
| --- | --- | --- | --- | --- | --- |
| **Donor** |  |  |  |  | < 0.001 |
| Deceased | 154 (96.2%) | 4 (80.0%) | 11 (61.1%) | 169 (92.3%) |  |
| Living | 6 (3.8%) | 1 (20.0%) | 7 (38.9%) | 14 (7.7%) |  |
| **Recipient gender** |  |  |  |  | 0.772 |
| Female (%) | 94 (58.8%) | 3 (60.0%) | 9 (50.0%) | 106 (57.9%) |  |
| Male (%) | 66 (41.2%) | 2 (40.0%) | 9 (50.0%) | 77 (42.1%) |  |
| **Transplant rank** |  |  |  |  | 0.576 |
| 1 | 50 (31.2%) | 2 (40.0%) | 9 (50.0%) | 61 (33.3%) |  |
| 2 | 92 (57.5%) | 3 (60.0%) | 6 (33.3%) | 101 (55.2%) |  |
| 3 | 17 (10.6%) | 0 (0.0%) | 3 (16.7%) | 20 (10.9%) |  |
| 4 | 1 (0.6%) | 0 (0.0%) | 0 (0.0%) | 1 (0.5%) |  |
| **Pretransplant dialysis** |  |  |  |  | 0.871 |
| No (%) | 8 (5.0%) | 0 (0.0%) | 1 (5.6%) | 9 (4.9%) |  |
| Yes (%) | 152 (95.0%) | 5 (100.0%) | 17 (94.4%) | 174 (95.1%) |  |
| **Dialysis vintage (years)** |  |  |  |  | 0.128 |
| Mean (SD) | 5.8 (5.9) | 6.1 (4.1) | 8.9 (8.9) | 6.1 (6.3) |  |
| Median (Q1, Q3) | 4.0 (2.2, 7.2) | 8.9 (3.0, 9.0) | 6.3 (4.2, 9.6) | 4.4 (2.2, 7.8) |  |
| Missing data | 8 | 0 | 1 | 9 |  |
| **BMI (kg/m2)** |  |  |  |  | 0.946 |
| Mean (SD) | 23.8 (5.0) | 24.5 (5.7) | 24.1 (5.5) | 23.9 (5.0) |  |
| Median (Q1, Q3) | 23.1 (20.1, 26.4) | 22.8 (19.6, 29.7) | 23.5 (20.3, 28.0) | 23.1 (20.1, 26.7) |  |
| **Recipient age years)** |  |  |  |  | 0.082 |
| Mean (SD) | 53.7 (13.3) | 46.5 (10.5) | 47.1 (13.5) | 52.9 (13.4) |  |
| Median (Q1, Q3) | 54.6 (42.7, 65.0) | 45.0 (38.7, 50.5) | 45.9 (40.3, 56.6) | 53.2 (42.1, 64.1) |  |
| **Cold ischemia time (minutes)** |  |  |  |  | 0.250 |
| Mean (SD) | 1199.9 (720.5) | 782.4 (147.1) | 1147.1 (831.8) | 1183.2 (723.5) |  |
| Median (Q1, Q3) | 1155.0 (770.0, 1657.5) | 800.0 (686.0, 870.0) | 1099.0 (482.5, 1818.8) | 1142.5 (714.0, 1631.2) |  |
| **Donor age (years)** |  |  |  |  | 0.849 |
| Mean (SD) | 55.7 (14.8) | 59.8 (11.8) | 55.6 (8.9) | 55.8 (14.2) |  |
| Median (Q1, Q3) | 57.5 (47.0, 66.0) | 55.0 (51.0, 65.0) | 56.0 (51.2, 61.0) | 57.0 (47.5, 66.0) |  |
| **Donor gender** |  |  |  |  | 0.565 |
| Female (%) | 66 (41.2%) | 3 (60.0%) | 9 (50.0%) | 78 (42.6%) |  |
| Male (%) | 94 (58.8%) | 2 (40.0%) | 9 (50.0%) | 105 (57.4%) |  |
| **HLA ABDRDQ mismatches** |  |  |  |  | 0.004 |
| 1 | 0 (0.0%) | 0 (0.0%) | 1 (5.6%) | 1 (0.5%) |  |
| 2 | 1 (0.6%) | 0 (0.0%) | 2 (11.1%) | 3 (1.6%) |  |
| 3 | 5 (3.1%) | 0 (0.0%) | 0 (0.0%) | 5 (2.7%) |  |
| 4 | 8 (5.0%) | 0 (0.0%) | 1 (5.6%) | 9 (4.9%) |  |
| 5 | 61 (38.1%) | 1 (20.0%) | 2 (11.1%) | 64 (35.0%) |  |
| 6 | 49 (30.6%) | 1 (20.0%) | 4 (22.2%) | 54 (29.5%) |  |
| 7 | 21 (13.1%) | 2 (40.0%) | 6 (33.3%) | 29 (15.8%) |  |
| 8 | 15 (9.4%) | 1 (20.0%) | 2 (11.1%) | 18 (9.8%) |  |
| **Induction therapy** |  |  |  |  | 0.978 |
| ATGAM | 1 (0.6%) | 0 (0.0%) | 0 (0.0%) | 1 (0.6%) |  |
| Basiliximab | 2 (1.3%) | 0 (0.0%) | 0 (0.0%) | 2 (1.1%) |  |
| ATG | 151 (98.1%) | 5 (100.0%) | 18 (100.0%) | 174 (98.3%) |  |
| Missing data | 6 | 0 | 0 | 6 |  |
| **ATG total dose (mg)** |  |  |  |  | 0.346 |
| Mean (SD) | 349.2 (509.8) | 343.0 (32.5) | 326.9 (118.6) | 346.8 (476.4) |  |
| Median (Q1, Q3) | 265.0 (250.0, 375.0) | 340.0 (325.0, 375.0) | 367.5 (250.0, 400.0) | 280.0 (250.0, 375.0) |  |
| Missing data | 8 | 0 | 0 | 8 |  |
| **Dialysis by D7 post-transplant** |  |  |  |  | 0.848 |
| No (%) | 130 (88.4%) | 4 (80.0%) | 15 (88.2%) | 149 (88.2%) |  |
| Yes (%) | 17 (11.6%) | 1 (20.0%) | 2 (11.8%) | 20 (11.8%) |  |
| Missing data (n) | 13 | 0 | 1 | 14 |  |
| **Tacrolimus trough level (ng/mL)** |  |  |  |  | 0.261 |
| Mean (SD) | 7.3 (3.5) | 5.3 (1.4) | 7.1 (3.7) | 7.2 (3.5) |  |
| Median (Q1, Q3) | 7.0 (5.0, 8.0) | 5.0 (4.0, 6.5) | 7.5 (5.2, 9.8) | 7.0 (5.0, 8.0) |  |
| Missing data (n) | 8 | 0 | 0 | 8 |  |
| **Bleeding complications** |  |  |  |  | 0.427 |
| No (%) | 1 (0.6%) | 0 (0.0%) | 0 (0.0%) | 1 (0.6%) |  |
| Yes (%) | 148 (96.1%) | 4 (80.0%) | 17 (94.4%) | 169 (95.5%) |  |
| Missing data (n) | 5 (3.2%) | 1 (20.0%) | 1 (5.6%) | 7 (4.0%) |  |
| **Peritransplant collection** |  |  |  |  | < 0.001 |
| Yes (%) | 14 (8.7%) | 2 (40.0%) | 1 (5.6%) | 17 (9.2%) |  |
| No (%) | 140 (90.9%) | 3 (60.0%) | 17 (94.4%) | 160 (90.4%) |  |
| Missing data (n) | 6 | 0 | 0 | 6 |  |
| **Infectious complications** |  |  |  |  | 0.166 |
| No (%) | 125 (81.2%) | 3 (60.0%) | 17 (94.4%) | 145 (81.9%) |  |
| Yes (%) | 29 (18.8%) | 2 (40.0%) | 1 (5.6%) | 32 (18.1%) |  |
| Missing data (n) | 6 | 0 | 0 | 6 |  |
| **BPAR at M12 post-transplant** |  |  |  |  | < 0.001 |
| No (%) | 155 (96.9%) | 5 (100.0%) | 13 (72.2%) | 173 (94.5%) |  |
| Yes (%) | 5 (3.1%) | 0 (0.0%) | 5 (27.8%) | 10 (5.5%) |  |

*Abbreviations*: KTx, kidney transplant; HLA, human leukocyte antigen; HLAc, HLA compatible; HLAi, HLA incompatible; pos, positive, neg, negative; CDC, complement-dependent cytotoxicity; BMI, body mass index; D, day; TGI, taux de greffons incompatibles; ATG, antithymocyte globulins; ABOi, ABO incompatible; BPAR, biopsy-proven acute rejection; M, month
